# Identification and Characterization of Citrus Concave Gum-Associated Virus Infecting Citrus and Apple Trees by Serological, Molecular and High-Throughput Sequencing Approaches

**DOI:** 10.3390/plants10112390

**Published:** 2021-11-05

**Authors:** Maria Minutolo, Maria Cinque, Michela Chiumenti, Francesco Di Serio, Daniela Alioto, Beatriz Navarro

**Affiliations:** 1 Dipartimento di Agraria, Università degli Studi di Napoli Federico II, 80055 Portici, Italy; minutolm@unina.it (M.M.); maria.cinque@unina.it (M.C.); 2 Istituto per la Protezione Sostenibile delle Piante, Consiglio Nazionale delle Ricerche, 70126 Bari, Italy; michela.chiumenti@ipsp.cnr.it (M.C.); francesco.diserio@ipsp.cnr.it (F.D.S.)

**Keywords:** apple rubbery wood virus 2, ARWV-2, CCGaV, coguvirus, concave gum disease, DAS-ELISA, DTBIA, next-generation sequencing, virus detection

## Abstract

Citrus concave gum-associated virus (CCGaV) is a negative-stranded RNA virus, first reported a few years ago in citrus trees from Italy. It has been reported in apple trees in the USA and in Brazil, suggesting a wider host range and geographic distribution. Here, an anti-CCGaV polyclonal antiserum to specifically detect the virus has been developed and used in a standard double antibody sandwich enzyme-linked immunosorbent assay (DAS-ELISA) that has been validated as a sensitive and reliable method to detect this virus both in citrus and apple trees. In contrast, when the same antiserum was used in direct tissue-blot immunoassay, CCGaV was efficiently detected in citrus but not in apple. Using this antiserum, the first apple trees infected by CCGaV were identified in Italy and the presence of CCGaV in several apple cultivars in southern Italy was confirmed by field surveys. High-throughput sequencing (HTS) allowed for the assembling of the complete genome of one CCGaV Italian apple isolate (CE-c3). Phylogenetic analysis of Italian CCGaV isolates from apple and citrus and those available in the database showed close relationships between the isolates from the same genus (*Citrus* or *Malus*), regardless their geographical origin. This finding was further confirmed by the identification of amino acid signatures specific of isolates infecting citrus or apple hosts. Analysis of HTS reads also revealed that the CE-c3 Italian apple tree, besides CCGaV, was simultaneously infected by several viruses and one viroid, including apple rubbery wood virus 2 which is reported for the first time in Italy. The complete or almost complete genomic sequences of the coinfecting agents were determined.

## 1. Introduction

Citrus concave gum-associated virus (CCGaV) was first identified in citrus trees in southern Italy [1], where it was found to be closely associated with symptoms of concave gum (CG), a citrus graft-transmissible virus-like disease which has been known for more than eighty years [2]. CCGaV is a flexuous and nonenveloped virus with a bipartite genome consisting of one negative-stranded (ns) RNA (RNA1), which codes for the viral RNA-dependent RNA polymerase (RdRp), and one ambisense RNA (RNA2), which contains two open reading frames (ORF) encoding the putative movement protein (MP) and the nucleocapsid protein (NP) [1]. The two ORFs in the RNA2 are in opposite orientation and separated by a long AU-rich intergenic region (IR) [1]. CCGaV is phylogenetically related to other members of the order *Bunyavirales* and has been recently classified as a representative species (*Coguvirus citri*) of the new genus *Coguvirus* of the family *Phenuiviridae* [3,4]. After being identified in citrus, CCGaV was reported to naturally infect apple, first in the USA [5] and then in Brazil [6]. Wright and colleagues [5] reported the presence of CCGaV in “Honeycrisp” apple trees grown in two orchards of central Washington State, and in several accessions from Spain, Italy, France, Australia and USA held at a germplasm collection of the Washington State University. In Brazil, CCGaV was detected in “Royal Gala” (RG) and “Mishima” (MI) grown in the field [6]. In both the USA and Brazil, CCGaV-infected apple trees were simultaneously infected by other viruses including apple rubbery wood virus 1 (ARWV-1) and ARWV-2, which are two negative ssRNA viruses classified in the genus *Rubodvirus* (family *Phenuiviridae*) and first reported in Germany, where they were found to be associated with wood rubbery disease [7]. Although CCGaV was identified in apple-decline-affected trees, the presence of other coinfecting viruses in these plants hampered the drawing of any conclusions on its role in eliciting any disease in apple [5,6].

Citrus trees are also natural hosts of another virus closely related to CCGaV, Citrus virus A (CiVA) [8], which has been also classified in a new species (*Coguvirus eburi*) of the genus *Coguvirus* [4]. CiVA was not associated with trunk (CG or cristacortis, CR) and leaf symptoms in citrus [8]. However, it has been recently associated with symptoms of impietratura, a disease inducing spotting and gumming in rind and albedo of fruits, on naturally infected grapefruit (*Citrus x paradisi* MacFad) trees grown in Greece [9] and with symptoms of oak leaf in indicator plants in both Greece and South Africa [9,10]. Akin to CCGaV, the host range of CiVA has been extended to a pome fruit tree species (*Pyrus communis* L.), although the virus pathogenicity in this host has not been assessed yet [11,12].

Molecular methods based on RT-PCR to specifically detect CCGaV and CiVA have been developed since early reports of these viruses [1,8]. Moreover, a multiplex RT-PCR protocol to identify and discriminate in a single reaction the two viruses in citrus has also been fixed recently [13], and an RT-qPCR has been applied for quantitative assays of CiVA in citrus fruits [9] and for the detection of CCGaV, CiVA and other negative-stranded RNA viruses [14]. Although these molecular methods allow detection of coguviruses, the availability of serological assays would strongly reduce the detection costs and provide new opportunity for large-scale surveys and for extensive tests requested by certification programs of vegetative propagation materials. An antiserum recognizing the CCGaV NP was previously developed based on synthetic peptides [1]. Although it was effective in trapping the virus and decorating it in transmission electron microscopy (TEM) assays [1], such an antiserum did not work properly when used in other serological tests, thus hindering its use for large-scale surveys in nurseries and or in the field.

Here, we report the development of a novel CCGaV-specific polyclonal antiserum against the CCGaV NP and show its sensitivity and reliability to specifically detect CCGaV by serological methods. Using this antiserum, the first CCGaV isolates infecting apple in Italy were identified. The specificity, sensitivity and reliability of the serological assays were validated and compared with RT-PCR-based methods both in citrus and apple. Field surveys confirmed the presence of CCGaV in several citrus species and in several apple cultivars grown in southern Italy. Further studies based on high-throughput sequencing (HTS) revealed the full-length genomic sequence of CCGaV from an Italian apple isolate, and together with traditional molecular approaches, allowed the assessment of the sequence variability of this virus in citrus and apple. Moreover, HTS approaches allowed identification of several other coinfecting viruses, including apple rubbery wood virus 2, which is reported for the first time in Italy.

## 2. Results

### 2.1. CCGaV Is Specifically Detected by Standard Double-Antibody Sandwich Enzyme-Linked Immunosorbent Assay (DAS-ELISA) Using a Polyclonal Antiserum in Both Citrus and Apple Trees

A rabbit polyclonal antiserum (CCGaV-ab1) against the N-terminal region (amino acids 1 to 119) of the CCGaV NP was generated and used to develop a DAS-ELISA protocol, in collaboration with Agritest. In selecting the protein region to be used to develop an antiserum specific for CCGaV, the possibility of cross-reaction of polyclonal antibodies with the NP of the closely related coguvirus CiVA, also infecting citrus [13], was taken into consideration. Indeed, the amino acid identity between the N-terminal region (amino acids 1 to 119) of the NP of the two viruses was only 37.29%, much lower than the identity in the rest of the protein (73.16%). Therefore, the use of a more divergent fragment would increase the expected specificity of the antiserum.

Several citrus host species previously tested for CCGaV and CiVA [13] were selected for this preliminary step. Reliability of the DAS-ELISA protocol was first tested by comparing the DAS-ELISA with the RT-PCR assay based on the primers CG.15 and CG.20 (Table S1). A total of 64 citrus trees of several species, including *C. clementina, C. limon, C. paradise, C. reticulata* and *C. sinensis,* were tested, with results obtained by DAS-ELISA completely matching with those based on the RT-PCR assay (Table 1). A total of 30 trees were infected by CCGaV, while 34 tested negative. Sixty-three of these samples were also tested by RT-PCR using the primer pair Ka-1/Ka-3 specific for coguvirus CiVA. Twenty trees were infected by CiVA, four of which were simultaneously infected by CCGaV, while 19 trees tested negative to both viruses (Table 1). Importantly, all the citrus trees infected by CiVA always tested negative to DAS-ELISA (Table 1), thus indicating no cross-reaction between the CCGaV-ab1 antiserum and the closely related CiVA. Moreover, as expected, positive signals were obtained from the four samples which were infected by both CiVA and CCGaV, thus confirming the absence of interference of the former on the detection of the latter virus by DAS-ELISA when the antiserum CCGaV-ab1 was used. Interestingly, these results were confirmed when the DAS-ELISA test was repeated in February, May, June, August, October and November, thus showing the possibility of also effectively using this serological assay during a nonvegetative growth period. Nevertheless, the best performance of the assay was obtained during the period of vegetative growth of the trees (May–June).

During the survey, symptoms on the trunk were also registered, discriminating plants with typical symptoms of CG and/or CR from those not showing these symptoms. Interestingly, CCGaV was almost exclusively detected in plants showing CG and CG plus CR symptoms, thus confirming data previously reported on the close association of this virus with concave gum symptoms in southern Italy (Campania region) [1] (Figure S1). In contrast, CiVA was not associated with any of the trunk symptoms considered in this study, thus confirming previous results [8] (Figure S2).

Extending DAS-ELISA assay to some apple trees grown in southern Italy (Campania region), CCGaV was also identified. The CCGaV infection was confirmed by RT-PCR and sequencing of the amplification products and by high-throughput sequencing (see below), thus providing the first evidence of the presence of this virus in apple trees grown in Italy. Based on these preliminary results, the DAS-ELISA protocol developed for citrus trees was applied in a survey to test 88 apple trees of several cultivars grown in orchards, in a germplasm collection or in a garden center germplasm in southern Italy (Campania region). Symptomless trees and trees showing trunk symptoms such as cracking (BCr), scaling (BS) and graft incompatibility (GI) were included in the survey. All the samples were also tested by RT-PCR to confirm the results obtained by the serological method. Twelve out the 88 assayed samples tested positive to both DAS-ELISA and RT-PCR (Table 2), with no sample testing positive to only one of the two assays, thus showing the reliability of the serological method to detect CCGaV in apple trees. The presence of this coguvirus was confirmed in globally distributed (Fuji, Gala, Golden) and local (San Nicola and Sergente) apple varieties (Table 2). These results were obtained by assaying the same trees in May, October and February, using young leaves, old leaves and buds, respectively (data not shown). No association was found between CCGaV and the trunk symptoms taken into consideration during the survey (Table 2). These data conclusively showed that CCGaV infects apple trees in Italy, although it does not seem to be responsible for a specific trunk alteration in this host.

To further assess sensitivity of the DAS-ELISA, a CCGaV-infected sweet orange (cv. Tarocco), a clementine, a lemon, and an apple (cv. Gala) tree and corresponding noninfected (NI) controls were selected to test the detection dilution limit of the assay. The test was conducted in autumn (October 2020), diluting the extracts obtained from expanded leaves of each CCGaV-infected plant in two-fold steps from 1/10 to 1/640, using healthy leaf extracts as a diluent. All tests were performed at the same time. CCGaV was efficiently detected up to a dilution of 1/320 in all the tested plants. The optical density (OD) values and the ratio with respect to the negative control (I/NI) were stronger in lemon with respect to the other species (Table 3). Apple was the host species generating the lowest colorimetric signals, likely because the leaves were already senescent when the survey was performed. Regardless, at a dilution of 1/320, the values from the infected apple sample were at least 10 times higher than those from the negative control (Table 3). A dilution of 1/320 would correspond to about 30 µg of infected tissue.

DAS-ELISA sensitivity was also evaluated by assaying different tissues from apple including petals, young and mature leaves, and buds collected in May, diluting the extracts as described in the previous experiments. The apple buds were the most performant tissues for this assay (Table 4). Although the best performance of the assay was achieved in May, CCGaV infection was also detected by DAS-ELISA in apple buds collected in February, during the period in which the plants are dormant (data not shown).

### 2.2. Direct Tissue Blot Immunoassay (DTBIA) Efficiently Detects CCGaV in Citrus but Not in Apple

**Figure 1:**
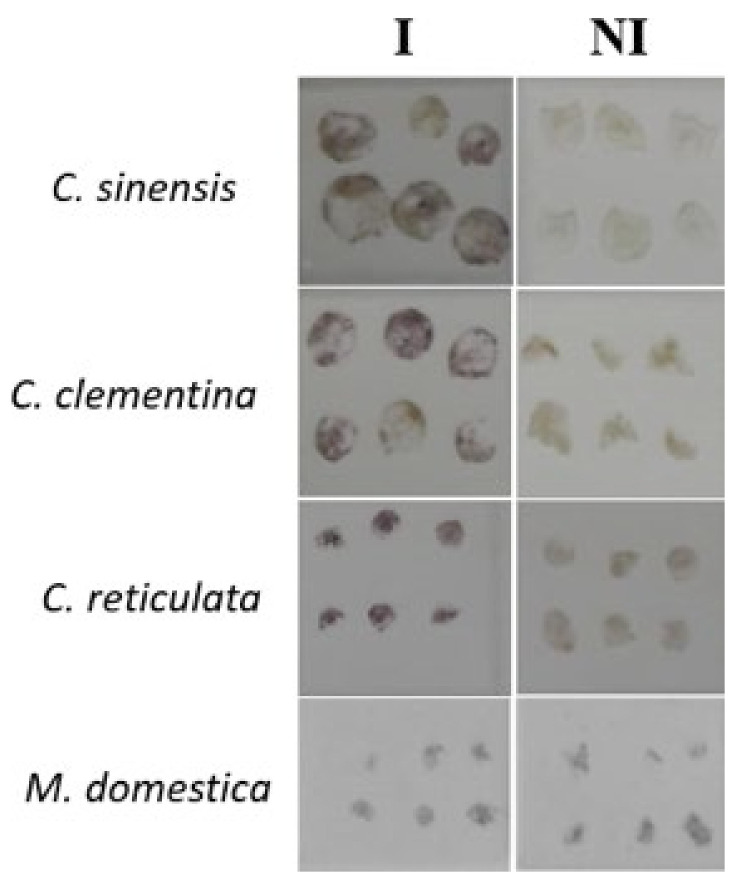
Direct tissue blot immunoassay (DTBIA) for CCGaV detection in citrus and apple trees. CCGaV-infected (**I**) and noninfected (**NI**) trees previously assayed by DAS-ELISA and RT-PCR (see Table 1 and Table 2) were tested. A representative subset of the results is shown, including I and NI tender shoot sections of *Citrus sinensis* and *C. clementina*, and leaf petiole sections of *C. reticulata* and *Malus domestica*. In each grid, six prints representative of different branches of a tree canopy are blotted. Purple coloration is visible on sections of CCGaV-infected *C. sinensis*, *C. clementina* and *C. reticulata* (MAD5, NAP1, and POR1 trees, respectively). No purple coloration is visible on sections of CCGaV-infected *M. domestica* (CE-c24 tree), indicating that the method is not efficient in apple. EB19, EB7, POR2 and CE-c22 trees were used as NI controls of *C. sinensis*, *C. clementina*, *C. reticulata* and *M. domestica,* respectively.

This technique is based on the direct blotting of plant tissues on the membranes and does not require extract preparation, thus allowing faster processing of samples. The assay has been previously successfully used for the detection of several viruses in several hosts [15]. In our case, DTBIA efficiently detected CCGaV in citrus samples, where an intense purple reaction was observed in imprinted infected tissues, while no purple reaction was visible in blots from negative samples (Figure 1). The test was extended to all the citrus samples tested by DAS-ELISA, providing reproducible results perfectly consistent with those obtained by DAS-ELISA and RT-PCR (Table 1). Therefore, DTBIA resulted a reliable method to detect CCGaV in citrus. In contrast, no reaction was observed in apple infected samples (Figure 1), probably due to the juiceless and/or the specific composition of the tissues used for printing the membranes. Therefore, DTBIA is not applicable to detect CCGaV in apple trees.

### 2.3. CCGaV Isolates Are Phylogenetically Correlated According to the Host

As a further step of our study, one CCGaV isolate from an apple tree (cv Fuji, isolate CE-c3), showing GI and BCr symptoms, was further characterized by high-throughput sequencing. Fifty-seven million reads were obtained. After trimming and filtering to remove low-quality reads, the reads were assembled into 36,430 contigs using SPAdes [16]. Three contigs of 6688, 1413 and 1304 nts showing 99% sequence identity with CCGaV RNA1 and RNA2, respectively, were identified (Table 5). Combining *de novo* assembling with the alignments to a reference sequence using Bowtie, the full-length genome of the CCGaV CE-c3 apple isolate was determined, with the RNA1 and RNA2 being composed of 6674 nt and 2704 nt, respectively (GenBank Accession numbers OK495690 and OK495691).

The sequence variability within the isolate was further investigated by mapping the reads on the CCGaV CE-c3 consensus sequence. No sequence variability was found in the RNA2 of this isolate. In contrast, four positions of the RNA1 (positions 629, 1826, 2524 and 5213) were polymorphic (C to T, T to A, A to G and A to T, respectively) in approximately 8.7 to 18.8% of the reads. No polymorphic positions were found within the RdRp core. These data support a low or almost no variability within the virus population from a single host.

**Figure 2:**
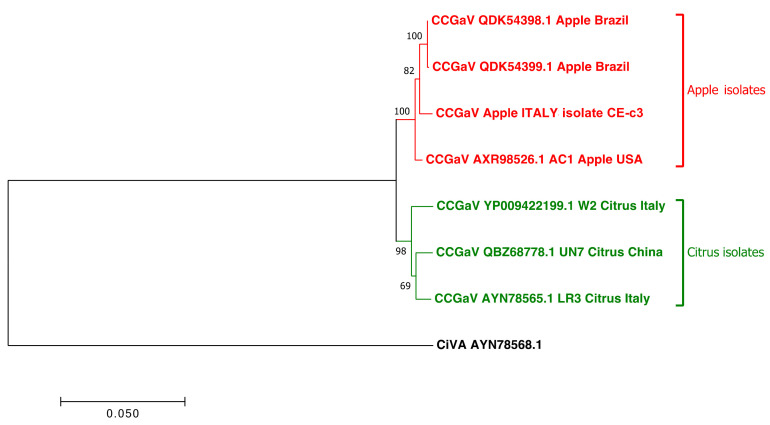
Phylogenetic tree generated using all the RNA1 genomic sequences of CCGaV available in databases and the Italian apple isolate CE-c3. Alignment of the nucleotide sequences with Clustal Omega was used for inferring the phylogenetic tree by the maximum likelihood method and adopting the best model JTT + I + F using MEGA7. Bootstrap probability values (1000 replicates) higher than 50% are shown at the branch nodes. Tree branches are proportional to the genetic distances, with the scale bar indicating substitutions per nucleotide site. Accession number, host, and country of origin of each CCGaV isolate are indicated at each branch tip. Citrus virus A (CiVA) was used as an outgroup.

**Figure 3:**
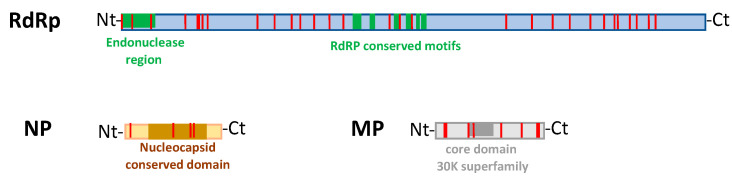
Position of amino acid signatures specific to CCGaV isolates infecting citrus and apple hosts (in red) identified in RdRp, NP and MP proteins. Conserved domains in each protein are indicated with different colors.

Pairwise alignments showed that RNA1 and RNA2 of the apple CE-c3 isolate have the best nucleotide and amino acid sequence-identity score (98–99%) in relation to the apple isolates from the USA and Brazil, rather than the citrus isolates from the same geographic origin (Table S2). Accordingly, a phylogenetic tree including all the RNA1 of CCGaV isolates previously reported showed that apple and citrus isolates clustered in two clearly different phylogenetic groups, regardless of their geographic origin (Figure 2). In addition, amino acid signatures specific of isolates infecting citrus or apple hosts were identified. They corresponded to thirty, four and ten polymorphic positions in the RdRp, NP and MP proteins of CCGaV, respectively (Figure 3 and Table S3). Those found in the RdRp protein are uniformly distributed and they do not affect the conserved amino acids of the endonuclease domain [H68-D80-PD97–98-(ExG109–111)-K128] and the five conserved motifs of the RdRp active site (pre-motif A and motifs A to D) (Figure 3). In the NP, only one out of the four changes between the apple and citrus isolates is located in the region selected to develop the specific antiserum used in the serological assays. However, this amino acid change does not affect the sensitivity of the DAS-ELISA assay. The host-specific changes found in the MP are unevenly distributed along the protein sequence, with five out of ten located in the C-terminal region and only one in the core domain of the MP belonging to the 30 K superfamily. Although the biological meaning of these host-specific amino acid signatures is currently unknown, they point to a possible host selective pressure on CCGaV.

**Figure 4:**
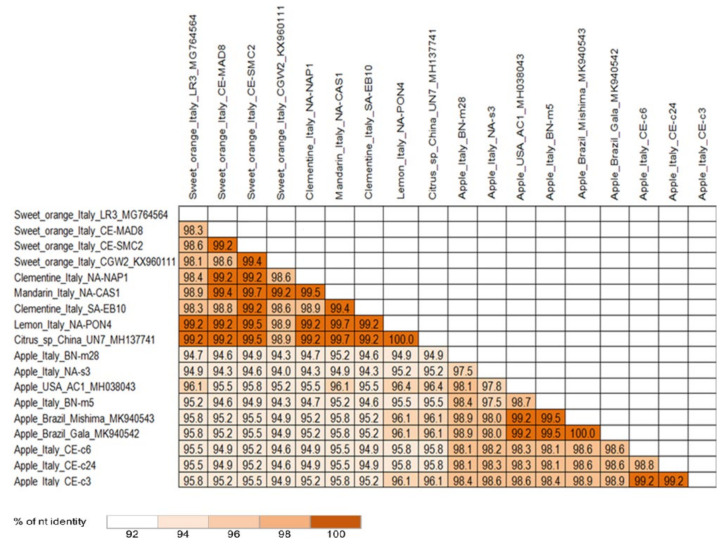
Pairwise nucleotide sequence-identity matrix of the nucleocapsid gene from apple and citrus Italian CCGaV isolates and from the other isolates in databases. The color of the cells indicates pairwise identity score (%) scaled from low (white) to high (brown). The host, the country of origin, the name and the identification number of each isolate are reported.

To further assess the sequence variability of CCGaV, the ORF encoding the NP (located in the RNA2) of the CCGaV from six citrus (SMC2, PON4, CAS1, MAD8, EB10, NAP1) and six apple (CE-c6, BN-m28, NA-s3, CE-c24, BN-m5 and CE-c3) trees grown in southern Italy were sequenced. Pairwise nucleotide analysis of the obtained sequences and those available in GenBank showed identity scores (94.3 to 96.4%) between the sequences from citrus and apple trees lower than those between isolates from the same host genus (98.1–100% between citrus isolates and 97.5–100% between apple isolates) (Figure 4), irrespective of the geographic origin. Moreover, a phylogenetic tree, generated using multiple alignments of the nucleotide sequences of the CCGaV gene encoding the NP from Italian isolates with those available in GenBank, confirmed the clustering of the CCGaV isolates according to their hosts rather than by geographic origin (Figure S3).

### 2.4. CCGaV Infects Apple in Association with Other Viruses

BlastN and BlastX analysis of the contigs generated from the HTS of the CE-c3 tree, analyzed against sequences of viruses and viroids in databases, allowed for the identification of apple stem pitting virus (ASPV), apple chlorotic leaf spot virus (ACLSV), ARWV-2, apple stem grooving virus (ASGV), and apple hammerhead viroid (AHVd), in addition to CCGaV (Table 5). Interestingly, the contigs covered the complete or almost complete genome of all the detected viruses and of AHVd (Table 5). Infection of the apple tree CE-c3 by the reported viruses was confirmed by RT-PCR (ASPV, ARWV-2, ASGV) or by DAS-ELISA (ACLSV). In contrast, several attempts to detect AHVd by RT-PCR failed, probably due to the low accumulation of the viroid in the infected tissues.

As far as we know, ARWV-2 was never reported previously in Italy. To further support this finding, infection by ARWV-2 was further investigated by testing additional apple trees from the same field where the CE-c3 tree was growing (Table S4). A total of forty trees, including the worldwide-cultivated cultivars Gala and Fuji (10 trees per cultivar) and the local cultivar Annurca (twenty trees), were assayed by RT-PCR. ARWV-2 was detected in eight plants of cultivar Fuji and in one of cultivar Annurca, while no one of the trees of cultivar Gala tested positive for this virus. The other three viruses coinfecting the CE-c3 tree (ASPV, ACLSV, ASGV) were reported to be widely spread worldwide and in Italy [17], and their presence in the tested apple cultivars was also confirmed by RT-PCR or by ELISA (in the case of ACLSV), with infection rates differing depending on the virus–cultivar combination taken into consideration (Table S4). Due to the simultaneous infection of tested samples by several viruses, it was not possible to associate CCGaV or any other of the detected viruses with the apple trunk/bark symptoms observed in some of plants.

HTS data provided additional information on all viruses identified in the CE-c3 isolate. Indeed, by the *de novo* assembling of the reads, the complete coding sequences of the RdRp, MP and NP genes of ARWV-2 Italian isolate CE-c3 were obtained. These genomic RNAs (GenBank accession numbers: OK562688, OK562689, OK562690) showed nucleotide sequence identities ranging from 96 to 98% with respect to the isolates previously reported from Canada and Germany [7] (Table 5). Regarding ASPV, eleven contigs with complete or almost complete genomic viral sequences (length from 8747 to 9373 nt) were assembled. These sequences shared a nucleotide identity ranging from 73% to 83% between them, indicating a high sequence variability within the CE-c3 isolate. These data are in agreement with previous studies on this virus in which significant levels of variability within individual hosts were observed [18]. The almost complete genome sequence of ASGV and ACLSV coinfecting the CE-c3 apple tree was also determined based on HTS data (Table 5). Although infection with AHVd was not confirmed by RT-PCR, a contig (coverage 782) consisting of more than one monomeric sequence of the viroid genome and showing the best nt identity (95%) with the Canadian AHVd isolate SD17_3-3 (ID: MK188692.1) was identified. Interestingly, the viroid associated with the CE-c3 apple tree diverged from AHVd isolates previously identified in southern Italy (Apulia region) in local apple cultivars [19].

## 3. Discussion

CCGaV is a virus which was identified a few years ago by high-throughput sequencing [1], and has been recently classified into the new genus *Coguvirus* of the family *Phenuiviridae*. In contrast with the other member of this family, which have a segmented genome composed of three or more RNAs, coguviruses have a bipartite genome composed of one negative-stranded RNA coding for the RdRp and a bipartite ambisense RNA encoding the NP and MP proteins. The initial identification of CCGaV in citrus [1] was followed by the identification of the virus in apple trees [5,6], thus extending its natural host range. However, while CCGaV has been associated with CG symptoms in citrus, whether it is associated with symptoms on the trunk or bark of apple has not been deeply investigated. CG has been reported to be a severe disease especially in young citrus trees [20]. However, the economic relevance of the disease is nowadays limited, likely as a consequence of its inclusion in certification programs of citrus vegetative propagation material, which rely on time- and cost-demanding bioassays.

Being a virus discovered only recently, the actual geographic distribution, as well as the natural transmission mechanisms of CCGaV, are almost unknown. At the beginning of this study, we retained that the availability of an antiserum specifically for detection of CCGaV would be helpful to further clarify the epidemiology of this virus because serological detection methods can be easily applied in large-scale surveys. Therefore, an antiserum against the N-terminal of the NP protein of CCGaV was generated, which allowed the identification of the first Italian isolates of this virus from apple trees.

The decision to use only the CCGaV N-terminal region of the NP, the most divergent protein region with respect to the corresponding protein of the closely related coguvirus CiVA, was appropriate for developing a specific antiserum against CCGaV which does not cross-react with CiVA. Indeed, the absence of colorimetric signals in citrus samples infected only by CiVA supports the absence of any cross-reaction between the polyclonal antibody and this virus. The specificity and sensitivity of the antiserum for the detection of CCGaV was tested and resulted to be very high when the DAS-ELISA protocol was applied to both citrus and apple samples. In the case of apple trees, several tissues including petals, young, mature and old leaves, and buds were shown to be appropriate as source plant material for the DAS-ELISA based on the novel antiserum, which efficiently detected CCGaV regardless of the seasons of the year in which the samples were taken in both citrus and apple. Importantly, this assay allowed for the identification of the virus also when using buds in wintertime, offering the possibility of detecting the virus in dormant apple plants. This opportunity extends the available time for performing field surveys and/or for testing vegetative propagation material.

Our study was integrated with data from a high-throughput sequencing approach that provided the full-length genome of CCGaV infecting an apple tree (CE-c3) grown in Italy, which allowed pairwise comparisons and phylogenetic analysis taking into consideration CCGaV isolates of different geographic origins, for which the complete genome was available. Interestingly, the CCGaV Italian apple isolate was more closely related to the other apple isolates from distant geographic areas, such as the USA and Brazil, than to the citrus isolates from southern Italy (Campania region). This finding was further corroborated by the analysis of the sequence variability of the NP coding region, which included additional twelve citrus and apple CCGaV isolates from southern Italy and those reported in other countries. In the pairwise identity matrix and in the phylogenetic tree generated with these sequences, the isolates from apple formed a cluster clearly distinct from the citrus isolates, regardless of the geographic origin. These data suggest the existence of a selective host pressure on CCGaV, which is further supported by the identification of amino acid signatures in the viral proteins, where certain polymorphic positions are occupied by specific residues depending on the infected host (apple or citrus). In the absence of an efficient bioassay, it is difficult to further investigate the biological meaning of these amino acid signatures. However, no amino acid changes were found in the endonuclease domain and the conserved motifs of the RdRp active site or in the core domain of the MP belonging to 30 K superfamily, supporting that their functionality is preserved in both group of isolates. Interestingly, in the region of the NP used to develop the specific CCGaV antiserum, only one nucleotide change in the isolates from apple was found with respect to those from citrus trees. Regardless, this change did not interfere with the capability of the antiserum to specifically recognize the virus, as shown by the high sensitivity of the DAS-ELISA test observed with both citrus and apple samples. Interestingly the novel antiserum efficiently detected CCGaV when the DTBIA method was applied to citrus isolates, thus providing a fast and economic alternative to the DAS-ELISA test for large-scale surveys. In contrast, DTBIA was not effective in the case of apple trees.

HTS data also showed that the apple tree CE-c3 was simultaneously infected by four viruses and one viroid, besides CCGaV. Simultaneous infection by one or more of these infectious agents was further confirmed by RT-PCR in several apple cultivars, thus showing a complex situation that impaired any association with a single virus or a mixed population with the symptoms observed in the tested plants. Although the infection by AHVd of the cultivar Fuji in southern Italy was not confirmed by RT-PCR, HTS data suggested that viroid isolates different from those reported previously in another Italian region (Apulia region) [19] are present in Italy, suggesting an independent origin of the diverging isolates. Importantly, the HTS approach allowed us to identify, for the first time in Italy, ARWV-2, a virus previously reported in Canada, Germany, USA, Brazil and China [5,6,7,21] in apple, and in China and South Africa in pear [22,23], and to determine the complete coding sequences of the genome of this virus.

The biological relationships between the citrus and apple CCGaV isolates are still unclear, and questions on whether and how the virus may be transferred between these hosts remain unanswered. No animal vector has been reported for CCGaV, and whether a fungus could be involved is not known and remains a seminal, open question. Our study, joining serological and molecular approaches, including HTS, provided interesting information on viruses infecting apple trees. On the one hand, the HTS approach was valuable for the molecular characterization of CCGaV and of the coinfecting viruses and viroid in apple. On the other hand, serological methods reported here will facilitate wide screening of propagation material and field surveys that may allow the identification of CCGaV in both citrus and apple trees, and possibly in other hosts, helping to further understand the epidemiology of this virus.

## 4. Materials and Methods

### 4.1. Sampling

Samples of different citrus species from 64 trees (Table 1), were collected in several citrus-growing areas of Campania in 2019 (February, May, June, August, October, November). Comparable numbers of representative samples showing CG or CR symptoms or no evident alterations on the trunk were selected. In addition, six trees displaying both CR and CG were included in the survey.

In 2019 (May and October) and 2020 (February), 88 samples of different apple varieties were collected from several orchards, a garden center, and a germplasm collection. Twenty-seven of these trees were symptomless (AS), 14 showed bark cracking (BCr), 32 graft incompatibility (GI), six bark scaling in the trunk and main branches (BS), one BCr and BS, and eight BCr and GI.

### 4.2. DAS-ELISA

Plant extracts were prepared by grinding 1.0 g tissue (buds, petals, leaves) and blending them in 10 volumes of 1× PBS (8 mM Na_2_HPO_4_, 1.5 mM KH_2_PO_4_, 2.7 mM KCl, 0.14 M NaCl, 3 mM NaN_3_, pH 7.4) containing 0.05% Tween 20, and 20 g/L polyvinyl pyrrolidone (PVP-24000) and 20 g/L defatted milk (extraction buffer). The plates (Microlon 96 W high binding; Greiner, Frickenhausen, Germany) were coated with 100 μL of the rabbit polyclonal antiserum anti-CCGaV (CCGaV-ab1) diluted 1:1000 in standard carbonate coating buffer, incubated at 37 °C for 2 h and washed five times with PBS-T (1× PBS plus 0.05% Tween 20). Extracts (100 μL) were added to the plate wells. After overnight incubation at 4 °C, the plates were washed as previously described and 100 μL of alkaline phosphatase-conjugated CCGaV-IgG diluted (1:1000) in conjugate buffer [1× PBS plus 0.05% Tween 20, 20 g/L polyvinyl pyrrolidone (PVP-24000) and 20 g/L defatted milk] was added and incubated for 2 h at 37 °C. Plates were washed and incubated with 100 μL of substrate (1 mg/mL of p-nitrophenyl phosphate in substrate buffer) at room temperature. ODs were measured at 405 nm using a microtiter plate reader (EL × 800; Bio-Tek Instruments, Winooski, VT, USA) after 30 min of incubation with the substrate. A sample was considered virus-positive if its OD was at least three times of the mean OD of the negative controls (noninfected trees). The same procedure was used for the detection of ACLSV in apple trees using the commercially available ELISA reagents (Bioreba AG, Reinach, Switzerland).

### 4.3. Direct Tissue Blot Immunoassay (DTBIA)

The test was performed by transversely cutting six tender tissues (young shoots or leaf petioles chosen depending on tissue availability on the tree species) from each tree and gently pressing the freshly cut surface on nitrocellulose (NC) membranes with a pore size of 0.45 µm (Bio-Rad, Hercules, CA, USA). The prints were air-dried for few minutes and incubated overnight at 4 °C in a blocking solution [10 g/L bovine serum albumin (BSA) in distilled water]. Membranes were incubated for 3 h at room temperature with alkaline phosphatase-conjugated CCGaV-IgG (CCGaV-ab2), diluted 1:1000 in 1× PBS (8 mM Na_2_HPO_4_, 1.5 mM KH_2_PO_4_, 2.7 mM KCl, 0.14 M NaCl, 3 mM NaN_3_, pH 7.4). After three washes of 5 min each with PBS-T (PBS plus 0.05% Tween 20), the AP activity was detected with the chromogenic substrate BCIP/NBT (Sigma-Aldrich, Saint Louis, MI, USA) according to the manufacturer’s instructions. After 5–10 min, the enzyme–substrate reaction was stopped by washing the membrane in distilled water. A purple–violet coloration on sections revealed the presence of CCGaV infection.

### 4.4. RNA Extraction and RT-PCR 

Total RNA was extracted from 200 mg of leaf tissues and reverse transcribed as reported previously by Minutolo et al. (2020). PCR amplification was carried out in 25 µL of reaction mixture using 1µL of cDNA, 1 U GoTaq Hot Start Polymerase (Promega, Mad- ison, WI, USA) and specific primers for each tested virus at a final concentration of 0.2 μM (Table S1). The cycling conditions were 94 °C for 3 min, 32 cycles of 94 °C for 30 s, 55 °C for 30 s, 72 °C for 30 s, and a final extension at 72 °C for 7 min. PCR reaction products were visualized by UV light, on 1.5% agarose gel stained with ethidium bromide. Nucleic acids enriched in double-stranded RNAs (dsRNAs) from apple leaf tissues were obtained with buffer-saturated phenol, and partitioned by chromatography on nonionic cellulose CF-11 (Whatman, Maidstone, UK) [1] and used for high-throughput sequencing.

### 4.5. High-Throughput Sequencing (HTS) and Bioinformatic Analysis

dsRNA-enriched nucleic acid preparations, digested with DNase turbo (Ambion, Foster City, CA, USA), and ribosome-depleted with Ribo-Zero Plant leaf kit (Illumina, San Diego, CA, USA), were used for generating an RNA-seq cDNA library according to the standard NEBNext Ultra II RNA Library Prep Kit for Illumina (New England Biolabs, Ipswich, MA, USA). Pair-end sequencing (2 × 150) of the library was performed on a NovaSeq 6000 sequencing system (Illumina). After trimming and quality control, the reads were *de novo* assembled in contigs using SPAdes [16]. The contigs were subsequently screened by BLASTN and BLASTX against virus and viroid sequences in the database. Bowtie software was used for alignment of HTS reads on virus/viroid sequences [24].

### 4.6. Phylogenetic Analyses

The complete ORF coding for the NP plus a short flanking sequence was amplified by RT-PCR (as described before) using one pair of specific oligonucleotides (CG-CP6/CG-CP7) for citrus samples, while two primer pairs (CG-CP1/CG-CP3 and CG-CP4/CG-CP5) (Table S1) generating two overlapping fragments covering the region of interest were used for apple samples. PCR amplicons were eluted from the agarose gel and both DNA strands were directly sequenced by Sanger technology using the same primers as used for PCR amplification. The obtained consensus sequences (GenBank accession numbers OK626278 to OK626288) were used for variability and phylogenetic analysis, taking into consideration the sequences obtained in this study and those available in the GenBank database. Multiple sequence alignments were generated by Clustal Omega [25] or Muscle [26]. Phylogenetic trees of the full-length RNA 1 or NP gene were inferred by maximum-likelihood method (ML) (1000 bootstrap replicates) with the best-fit model JTT + I + F and T92 + I, respectively.

## Figures and Tables

**Table 1:** Results of citrus field survey for detection of CCGaV, using different detection methods, and CiVA using RT-PCR.

Species	Symptoms	Number of Trees	Isolate Name(s)	CCGaV	CCGaV	CCGaV	CiVA
				RT-PCR	DAS-ELISA	DTBIA	RT-PCR
*Citrus clementina*	AS	1	EB8	NEG	NEG	NEG	NEG
*Citrus clementina*	CG	3	EB10, NAP1, NAP2	POS	POS	POS	NEG
*Citrus clementina*	CG	1	EB17	POS	POS	POS	POS
*Citrus clementina*	CR	2	EB7, 14O	NEG	NEG	NEG	NEG
*Citrus limon*	AS	1	PON4	POS	POS	POS	NT
*Citrus limon*	AS	1	EB38	NEG	NEG	NT	NEG
*Citrus limon*	CG	1	EB13	POS	POS	POS	NEG
*Citrus limon*	CG	1	EB5	POS	POS	NT	NEG
*Citrus paradisi*	CR	1	MAD16	NEG	NEG	NEG	POS
*Citrus reticulata*	AS	1	14E	NEG	NEG	NEG	POS
*Citrus reticulata*	AS	3	MAD20, NAP3, POR3	NEG	NEG	NEG	NEG
*Citrus reticulata*	CG	1	MAD17	POS	POS	POS	NEG
*Citrus reticulata*	CG	1	CAS1	POS	POS	NT	NEG
*Citrus reticulata*	CG + CR	2	MAD19, POR1	POS	POS	POS	NEG
*Citrus reticulata*	CG + CR	1	NAP4	POS	POS	NT	POS
*Citrus reticulata*	CG + CR	1	14H	POS	POS	POS	POS
*Citrus reticulata*	CR	6	14A, 14I, 14N,14R, 14S, SAA20	NEG	NEG	NEG	POS
*Citrus reticulata*	CR	3	MAD21, MAD22, POR2	NEG	NEG	NEG	NEG
*Citrus reticulata*	CR	1	SMC2	POS	POS	NT	NEG
*Citrus sinensis*	AS	9	14D, 14M, EB15, EB16, EB3, EB33, I13, MAD4, MAD9	NEG	NEG	NEG	NEG
*Citrus sinensis*	AS	4	EB19, EB20, EB30, EB4	NEG	NEG	NEG	POS
*Citrus sinensis*	CG	1	14L	POS	POS	POS	POS
*Citrus sinensis*	CG	11	EB1, EB2, EB14, EB18, EB21, EB22, EB24, EB25, ER1, MAD5, I11	POS	POS	POS	NEG
*Citrus sinensis*	CG	1	POR4	POS	POS	NT	NEG
*Citrus sinensis*	CR	3	14Q, MAD3, SAA16	NEG	NEG	NEG	POS
*Citrus sinensis*	CR	1	SMC3	POS	POS	NT	NEG
*Citrus sinensis*	CG + CR	1	MAD1	POS	POS	POS	POS
*Citrus sinensis*	CG + CR	1	MAD8	POS	POS	POS	NEG

**Table 2:** Field survey of CCGaV in apple using RT-PCR and DAS-ELISA.

Apple cv	Symptoms *	Number of Trees	Isolate Name(s)	RT-PCR	DAS-ELISA
Agostinella rossa	AS	1	BN-b9	NEG	NEG
Annurca	BCr	1	BN-b29	NEG	NEG
Annurca	AS	8	BN-b31, BN-b27, BN-b27a, BN-b27b, BN-b27c, BN-b27d, BN-m25, NA-s1	NEG	NEG
Annurca	GI	20	CE-c11 to CE-c20, CE-c31 to CE-c40	NEG	NEG
Arancio	BCr	1	BN-b15	NEG	NEG
Austina	AS	1	BN-b6	NEG	NEG
Bianca di Grottolella	BCr	1	BN-b12	NEG	NEG
Bella del giardino	AS	1	BN-m6	NEG	NEG
Calvilla estiva	AS	1	BN-m1	NEG	NEG
Cancavone	BCr	1	BN-b24	NEG	NEG
Cusanara	BCr	1	BN-b36	NEG	NEG
D’Andrè	AS	1	BN-m22	NEG	NEG
Fragola	BCr, BS	1	BN-b4	NEG	NEG
Fragola	BCr	1	BN-b28	NEG	NEG
Fuji	GI, BCr	4	CE-c3, CE-c6, CE-c7, CE-c8	POS	POS
Fuji	GI, BCr	4	CE-c1, CE-c4, CE-c5, CE-c9	NEG	NEG
Fuji	GI	2	CE-c2, CE-c10	NEG	NEG
Gala	AS	1	BN-m5	POS	POS
Gala	GI	4	CE-c23, CE-c24, CE-c26, CE-c30	POS	POS
Gala	GI	6	CE-c21, CE-c22, CE-c25, CE-c27, CE-c28, CE-c29	NEG	NEG
Gelata	AS	3	BN-m2, BN-m19, BN-m26	NEG	NEG
Golden	AS	2	BN-m3, NA-s2	NEG	NEG
Golden	AS	1	NA-s3	POS	POS
Lazzarola	BS	1	BN-b11	NEG	NEG
Limoncella	AS	2	BN-m13, BN-m18	NEG	NEG
Melone	BCr	1	BN-b26	NEG	NEG
Monaca	AS	1	BN-b7	NEG	NEG
Rondella	AS	1	BN-m4	NEG	NEG
San Francesco	BS	1	BN-b20	NEG	NEG
San Nicola	BCr	2	BN-b17, BN-m27	NEG	NEG
San Nicola	AS	1	BN-m28	POS	POS
Sergente	BCr	1	BN-b1	POS	POS
Sergente	BCr	1	BN-b18	NEG	NEG
Suricillo	BS	2	BN-b14, BN-b16	NEG	NEG
Tenerella	AS	1	BN-b13	NEG	NEG
Trumuntana	BCr	1	BN-b10	NEG	NEG
Tubiona	BCr	1	BN-b8	NEG	NEG
Vivo	BS	1	BN-b19	NEG	NEG
Zampa di Cavallo	BS	1	BN-b2	NEG	NEG
Zitella	BCr	1	BN-b25	NEG	NEG
Zitella	AS	1	BN-m23	NEG	NEG

* AS, asymptomatic; BCr, bark cracking; BS, bark scaling; Gl, graft incompatibility.

**Table 3:** Sensitivity of the DAS-ELISA test in different citrus species and apple using the antiserum against NP protein of CCGaV.

Dilution ^a^		Lemon		Clementine		Sweet Orange		Apple	
		OD (SD) ^b^	I/NI ^c^	OD (SD) ^b^	I/NI ^c^	OD (SD) ^b^	I/NI ^c^	OD (SD) ^b^	I/NI ^c^
1/10	1 mg	1.653 (0.114)	551	1.196 (0.068)	149	0.818 (0.022)	818	0.301 (0.030)	301
1/20	500 µg	1.306 (0.008)	435	0.894 (0.080)	112	0.610 (0.006)	610	0.168 (0.012)	168
1/40	250 µg	0.934 (0.124)	311	0.523 (0.089)	65	0.326 (0.005)	326	0.081 (0.005)	81
1/80	125 µg	0.673 (0.144)	224	0.328 (0.098)	41	0.184 (0.006)	184	0.042 (0.003)	42
1/160	62.5 µg	0.365 (0.028)	122	0.143 (0.027)	18	0.110 (0.014)	110	0.019 (0.003)	19
1/320	31.3 µg	0.195 (0.023)	65	0.083 (0.030)	10	0.054 (0.006)	54	0.010 (0.003)	10
1/640	15.1 µg	0.100 (0.012)	33	0.040 (0.009)	5	0.0023 (0.001)	23	0.005 (0.004)	5
Negative control (NI)		0.003 (0.003)		0.008 (0.012)		0.001 (0.001)		0.001 (0.005)	

^a^ Crude extracts from infected tissues were diluted in two-fold steps from 1/10 to 1/640 using noninfected (NI) leaf extracts as diluent. The corresponding amounts of infected tissues for each dilution are reported on the right column. ^b^ Mean Optical Density (OD) at 405 nm for ten technical replicates measured after 30 min of incubation. In brackets is the standard deviation (SD). ^c^ Ratio between OD in CCGaV-infected (I) and OD in the control noninfected plant (NI).

**Table 4:** Sensitivity of DAS-ELISA test in different apple tissues using the antiserum against NP protein of CCGaV.

Dilution ^a^		Petals		Young Leaves		Mature Leaves		Buds	
		OD (SD) ^b^	I/NI ^c^	OD (SD) ^b^	I/NI ^c^	OD (SD) ^b^	I/NI ^c^	OD (SD) ^b^	I/NI ^c^
1/10	1 mg	1.076 (0.011)	1076	0.886 (0.132)	886	1.242 (0.092)	1242	1.824 (0.049)	1824
1/20	500 µg	0.685 (0.052)	685	0.461 (0.016)	461	0.752 (0.021)	752	1.249 (0.075)	1249
1/40	250 µg	0.301 (0.013)	301	0.238 (0.006)	238	0.366 (0.012)	366	0.665 (0.024)	665
1/80	125 µg	0.161 (0.027)	161	0.139 (0.004)	139	0.224 (0.011)	224	0.397 (0.037)	307
1/160	62.5 µg	0.081 (0.012)	81	0.060 (0.012)	60	0.106 (0.010)	106	0.182 (0.047)	182
1/320	31.3 µg	0.036 (0.001)	36	0.039 (0.006)	39	0.060 (0.013)	60	0.076 (0.009)	76
1/640	15.1 µg	0.017 (0.005)	17	0.019 (0.004)	19	0.025 (0.004)	25	0.036 (0.003)	36
Negative control (NI)				0.001 (0.002)					

^a^ Crude extracts from infected tissues were diluted in two-fold steps from 1/10 to 1/640 using noninfected (NI) leaf extracts as diluent. The corresponding amounts of infected tissues for each dilution are reported in the right column. ^b^ Mean Optical Density (OD) at 405 nm for ten technical replicates measured after 30 min of incubation. In brackets is the standard deviation (SD). ^c^ Ratio between OD in CCGaV-infected (I) and OD in the control noninfected plant (NI).

**Table 5:** Assembled contigs corresponding to full-length or almost-full-length genomic sequences of known viruses in the database.

Virus Name	Name of Contig	Contig Length (nt)	Contig Coverage	Genome Coverage	Closest Isolate (% of nt Identity)
Apple stem pitting virus	NODE_18	9471	9.142201	100%	LC533839.1 isolate GCSPV2 (80%)
Apple stem pitting virus	NODE_19	9372	6.513875	99.9%	KF321967.1 isolate Hannover (85.65%)
Apple stem pitting virus	NODE_20	9366	5.155808	100%	LC533839.1 isolate GCSPV2 (85.39%)
Apple stem pitting virus	NODE_21	9315	2.486410	100%	LC475150.1 isolate SG (81.5%)
Apple stem pitting virus	NODE_22	9309	2.486410	100%	LC475150.1 isolate SG (82.2%)
Apple stem pitting virus	NODE_23	9301	2.332535	99.8%	KY490039.1 isolate ASPV-XC (80.2%)
Apple stem pitting virus	NODE_24	9267	3.684906	100%	LC475150.1 isolate SG (81.9%)
Apple stem pitting virus	NODE_25	9250	3.971113	100%	LC475150.1 isolate SG (81.7%)
Apple stem pitting virus	NODE_26	9247	3.582944	100%	LC475150.1 isolate SG (82.4%)
Apple stem pitting virus	NODE_28	8894	3.582944	96.0%	LC475150.1 isolate SG (81.5%)
Apple stem pitting virus	NODE_30	8747	2.495947	93.8%	KF321967.1 isolate Hannover (82.9%)
Apple chlorotic leaf spot virus	NODE_34	7590	19.237599	100%	LC533838.1 isolate RRACV2 (84%)
Apple stem growing virus	NODE_42	6536	126.968872	100%	JX080201.1 isolate AC (98.1%)
Apple stem growing virus	NODE_43	6532	32.303380	100%	MF616381.1 isolate JLSG (91.0%)
Apple rubbery wood virus 2	NODE_36	7384	7.281452	100%	RNA1 MF062128.1, MW322828.1, MF062133.1, NC_055534.1, MF062144.1; isolates:982-11, FT146, 355-1, R12, R7 (98.1%)
Apple rubbery wood virus 2	NODE_1717	1557	96.438451	100%	RNA2 MF062146 isolate R7 (96.2%)
Apple rubbery wood virus 2	NODE_1467	1644	64.116112	100%	RNA3 MF062145 isolate R7 (97.18%)
Apple hammerhead viroid	NODE_11893	544	78.2487298	100%	MK188692.1 isolate SD17_3-3 (95.3%)
Citrus concave gum-associated virus	NODE_41	6688	5.300137	99.0%	RNA1 MH038042 isolate AC1 (99%)
Citrus concave gum-associated virus	NODE_2231	1413	67.451613	52.0%	RNA2 MH038043 isolate AC1 (99%)
Citrus concave gum-associated virus	NODE_2730	1304	52.79212	47.0%	RNA2 MK940543 isolate Mishima (99%)

## Data Availability

The data presented in this study are available in article and supplementary material.

## Supplementary Materials

The following are available online at https://www.mdpi.com/article/10.3390/plants10112390/s1. Figure S1: Analysis of the association between CCGaV infection and symptoms of concave gum (CG), cristacortis (CR), CG plus CR (CG+CR) or absence of symptoms (AS) in different citrus species tested in the field survey. The histogram shows the number of noninfected (-) and CCGaV-infected (+) trees for each citrus species and for the total trees analyzed, with the observed symptoms (CG, CG+CR, CR and AS) denoted by different colors. Figure S2: Analysis of the association between CiVA infection and the symptoms of concave gum (CG), cristacortis (CR), CG plus CR (CG+CR), or the absence of symptoms (AS) in different citrus species tested in the field survey. The histogram shows the number of noninfected (-) and CiVA-infected (+) trees for each citrus species and for the total trees analyzed, with the observed symptoms (CG, CG+CR, CR and AS) denoted by different colors. Figure S3: Phylogenetic tree of nucleoprotein (NP) gene sequences of the Italian citrus and apple isolates of citrus concave gum-associated virus (CCGaV) that have been characterized in this study and those of the other isolates from different countries available in GenBank. Sequence alignment was generated by MUSCLE and was used for the phylogenetic tree construction using the maximum-likelihood method (best model T92 + I) by MEGA X, with 1000 bootstrap replicates. Bootstrap probability values higher than 50% are shown at the branch nodes. Genetic distances are indicated below each branch. The host, the country of origin, the name, and the accession number of each CCGaV isolate are indicated at each branch tip. Citrus virus A (CiVA) was used as an outgroup. Table S1: Primers used in this study. Table S2: Pairwise nucleotide and amino acid identity scores (%) of CCGaV Italian apple isolate CE-c3 with CCGaV isolates in databases. Table S3: Amino acid-specific signatures of CCGaV of apple and citrus isolates. Table S4: Limited survey of the viruses identified by NGS in the CE-c3 apple tree (number of infected trees/number of total analyzed).
