# Supplementary material for: Identification and Characterization of Citrus Concave Gum-Associated Virus Infecting Citrus and Apple Trees by Serological, Molecular and High-Throughput Sequencing Approaches
Source: Plants (Basel). 2021 Nov 5;10(11):2390. doi: 10.3390/plants10112390 (PMC8625769; doi:10.3390/plants10112390)
Supplement: Supplementary file 1 [file plants-10-02390-s001.zip › Supplementary material_Plants_1452222_final version.pdf]

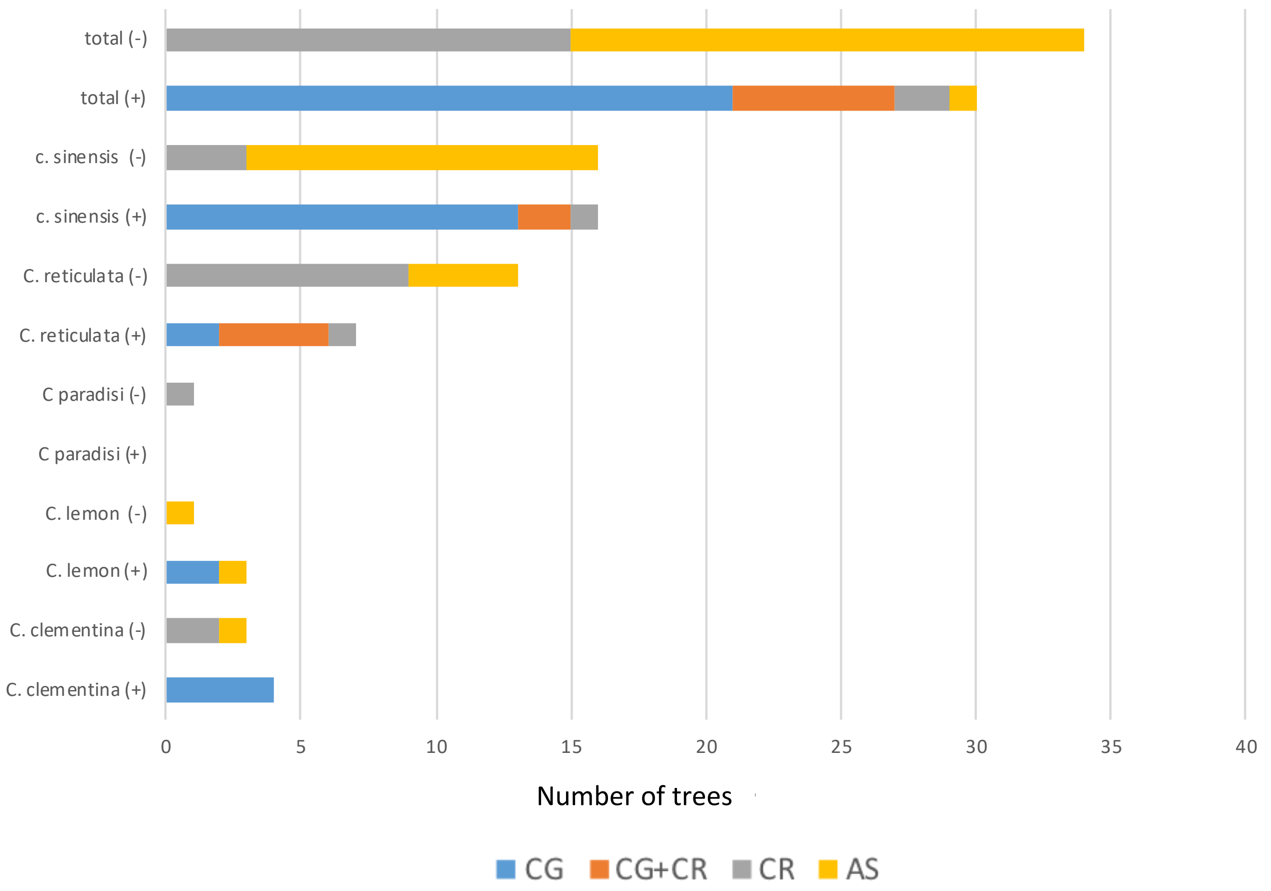

Figure S1 Analysis of the association between CCGaV infection and symptoms of concave gum (CG), cristacortis (CR), CG plus CR (CG+CR) or absence of symptoms (AS) in different citrus species tested in the field survey. The histogram shows the number of noninfected (-) and CCGaV-infected (+) trees for each citrus species and for the total trees analyzed, with the observed symptoms (CG, CG+CR, CR and AS) denoted by different colors

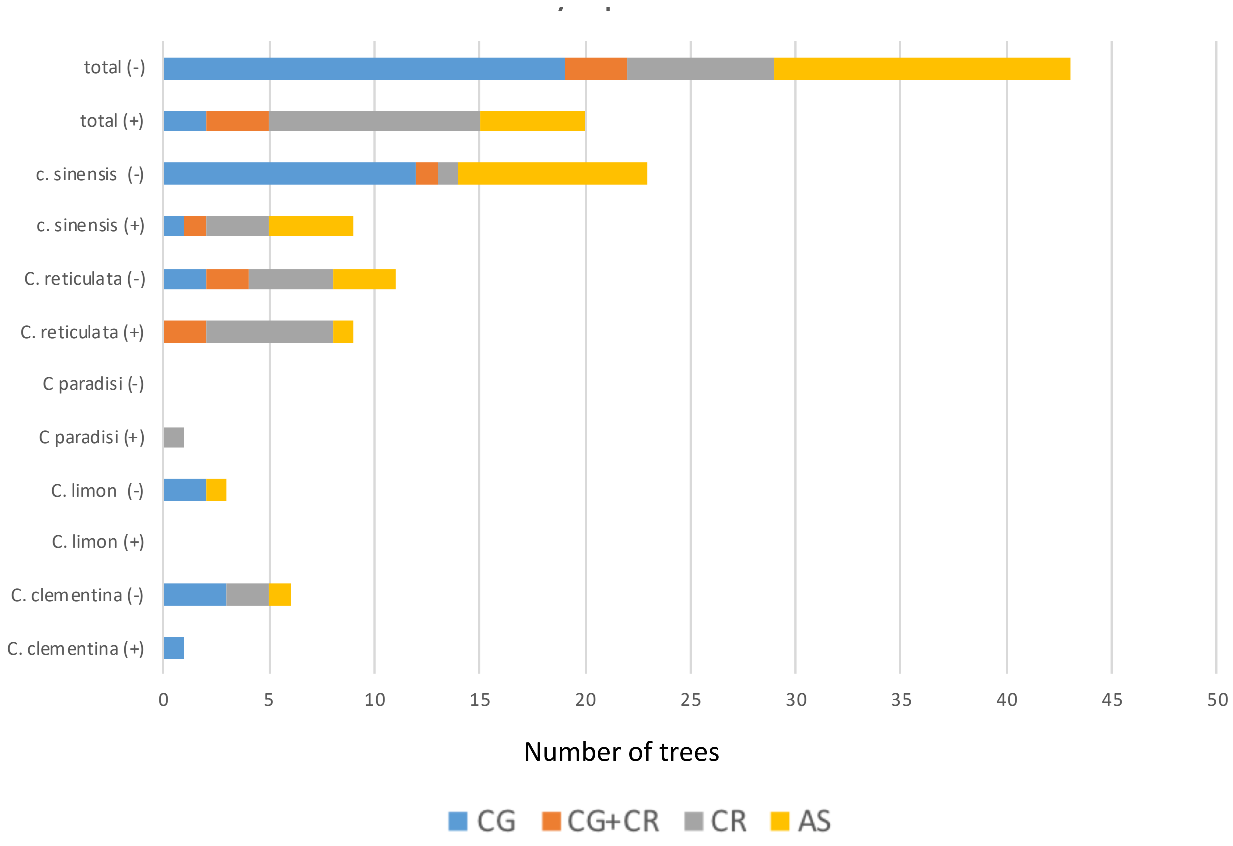

Figure S2 Analysis of the association between CiVA infection and the symptoms of concave gum (CG), cristacortis (CR), CG plus CR (CG+CR), or the absence of symptoms (AS) in different citrus species tested in the field survey. The histogram shows the number of noninfected (-) and CiVA-infected (+) trees for each citrus species and for the total trees analyzed, with the observed symptoms (CG, CG+CR, CR and AS) denoted by different colors.

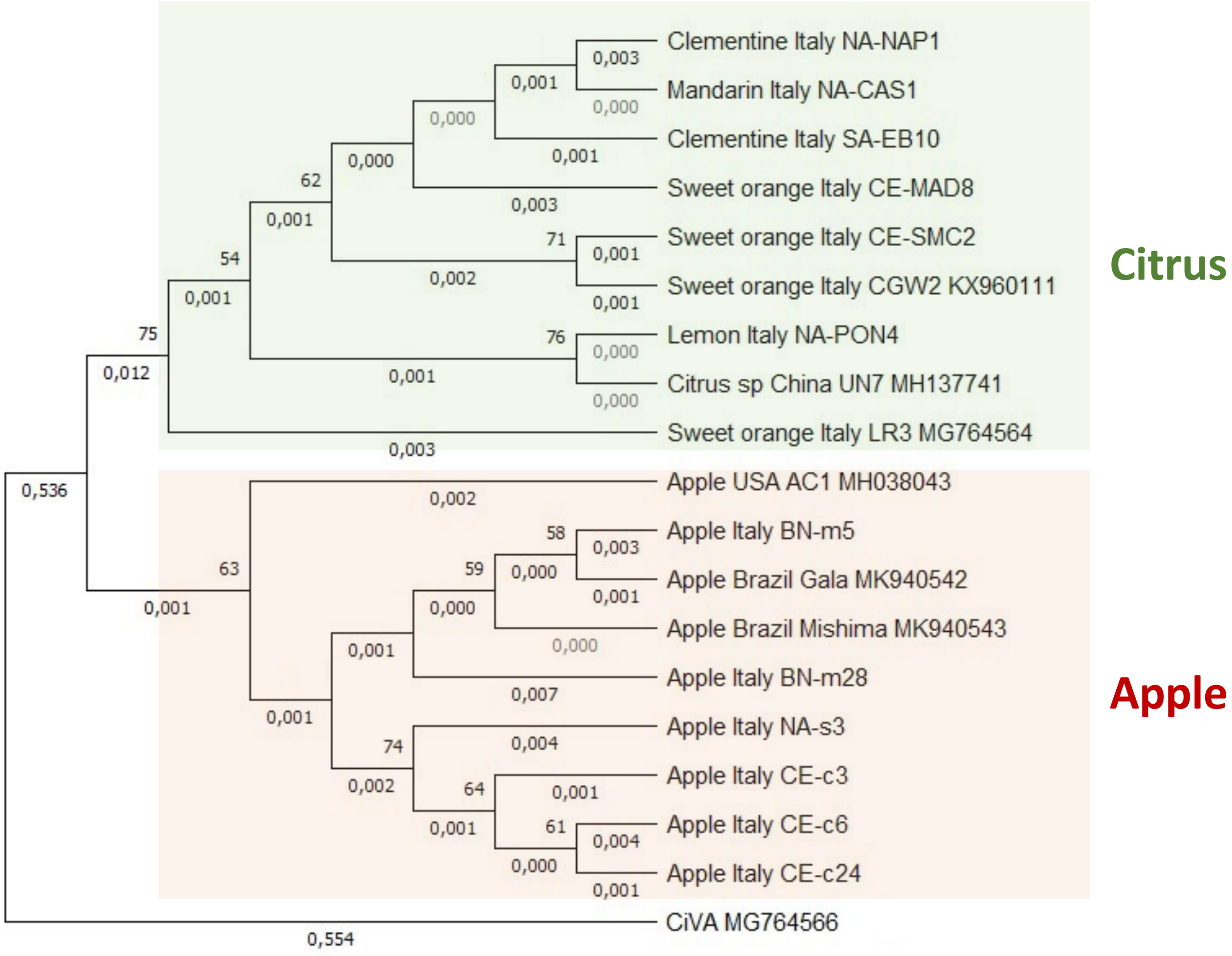

Figure S3 Phylogenetic tree of nucleoprotein (NP) gene sequences of the Italian citrus and apple isolates of citrus concave gum-associated virus (CCGaV) that have been characterized in this study and those of the other isolates from different countries available in GenBank. Sequence alignment was generated by MUSCLE and was used for the phylogenetic tree construction using the maximum-likelihood method (best model T92+I) by MEGA X, with 1000 bootstrap replicates. Bootstrap probability values higher than 50% are shown at the branch nodes. Genetic distances are indicated below each branch. The host, the country of origin, the name, and the accession number of each CCGaV isolate are indicated at each branch tip. Citrus virus A (CiVA) was used as an outgroup.

Table S1 Primers used in this study

Virus	Primer name	Sequence 5' to 3'	Used for	Reference
CCGaV	CG.15_For	AGGCTTTTCTTTCTTGTTCGGA	Survey	[1]
CCGaV	CG.20_Rev	CAAGAGAGGCAGTGGGAGAA	Survey	[1]
CiVA	Ka-1	TCCTGATGAAGTCTTAAGATCGC	Survey	[8]
CiVA	Ka-3	TTGCAGTAGTGAGAAGGGAGT	Survey	[8]
CCGaV	CG-CP1	AACCAAAAAGTTGGGCTCTG	variability NP apple	This study
CCGaV	CG- CP3	AAGGGATCCATTAATGAACACAA	variability NP apple	This study
CCGaV	CG-CP4	TTCCAAAGATGAAGGTATCACAGC	variability NP apple	This study
CCGaV	CG-CP5	GAACCCATAACTTTTGTTATCAACTC	variability NP apple	This study
CCGaV	CG-CP6	TCTAAAATGACAGATGGAAATATGCT	variability NP citrus	This study
CCGaV	CG-CP7	ACACATAGAACCCATAACTTTTGT	variability NP citrus	This study
ASPV	ASPF1CP	GGGTGTACTTTGAGGCAGTATT	Survey	Komorowska et al., 2010*
ASPV	ASPR3CP	GAGCGGATGCGGTACATCTGTAT	Survey	Komorowska et al., 2010*
ARWV-2	ARW2-F1	ATGTTGCATCACAGCTATTGGC	Survey	[6]
ARWV-2	ARW2-R1	ATTGTTCCATGCTGCCACAGAA	Survey	[6]
ASGV	ASGV-U	CCCGCTGTTGGATTTGATACACCTC	Survey	James, 1999**
ASGV	ASGV-2	GGAATTTCACACGACTCCTAACCCTCC	Survey	James, 1999**

*Komorowska, B.; Malinowski, T.; Michalczuk, L. Evaluation of several RT-PCR primer pairs for the detection of Apple stem pitting virus. *J. Virol. Methods*. **2010,** *168*, 242–247. https://doi.org/10.1016/j.jviromet.2010.04.024

James, D. A simple and reliable protocol for the detection of apple stem grooving virus by RT-PCR and in a multiplex PCR assay. *J. Virol. Methods* **1999, *83*, 1–9. https://doi.org/10.1016/s0166-0934(99)00078-6

Table S2 Pairwise nucleotide and amino acid identity scores (%) of CCGaV Italian apple isolate CE-c3 with CCGaV isolates in databases

Isolate	Host	Country	ID (RNA1; RNA2)	RNA1[a]	RNA2[a]	RdRp[b]	NP[b]	MP[b]
AC1	apple	USA	MH038042.1 ;MH038043.1	98.9	99.1	99.0	99.4	98.8
Gala	apple	Brazil	MK940540.1; MK940542.1	98.3	98.6	99.2	99.4	98.0
Mishima	apple	Brazil	MK940541.1; MK940543.1	98.3	98.3	99.1	99.7	98.0
CGW2	citrus	Italy	NC_035759.1; NC_035454.1	97.0	97.2	97.2	98.3	96.1
LR3	citrus	Italy	MG764563.1; MG764564.1	97.0	97.2	97.3	98.3	95.6
UN7	citrus	China	MH137740.1; MH137741.1	96.9	97.3	97.2	98.3	95.6

[a] nt identity score

[b] aa identity score

Table S3 Amino acid specific signatures of CCGaV of apple and citrus isolates

Protein	aa position	aa in Citrus	aa in Apple
NP	30	V	A
NP	188	N	S
NP	249	E	D
NP	340	N	S
MP	34	A	V
MP	36	I	T
MP	123	D	E
MP	142	V	I
MP	242	C	S
MP	319	N	K
MP	383	Q	H
MP	384	T	A
MP	388	K	R
MP	393	N	D
RdRp	8	V	I
RdRp	44	I	V
RdRp	124	V	L
RdRp	252	R	S
RdRp	288	R	K
RdRp	292	E	D
RdRp	307	F	Y
RdRp	343	V	I
RdRp	509	S	L
RdRp	577	V	I
RdRp	644	T	S
RdRp	673	F	Y
RdRp	728	D	N
RdRp	795	I	V
RdRp	835	C	F
RdRp	1010	K	T
RdRp	1047	A	T
RdRp	1094	I	T
RdRp	1446	S	L
RdRp	1544	I	V
RdRp	1590	I	T
RdRp	1677	I	T
RdRp	1763	E	V
RdRp	1805	L	I
RdRp	1851	T	S
RdRp	1855	I	V
RdRp	1908	D	E
RdRp	1927	V	I
RdRp	1976	Q	H

Table S4 Limited survey of the viruses identified by NGS in the CE-c3 apple tree (number of infected trees/number of total analysed)

Virus	cv. Gala	cv. Fuji	cv. Annurca
Apple rubbery wood virus 2	0/10	10/10	1/20
Apple stem grooving virus	0/10	10/10	15/20
Apple stem pitting virus	7/10	10/10	19/20
Apple chlorotic leafspot virus	0/10	8/10	19/20
